# Digital PCR improves the quantitation of DMR and the selection of CML candidates to TKIs discontinuation

**DOI:** 10.1002/cam4.2087

**Published:** 2019-04-04

**Authors:** Simona Bernardi, Michele Malagola, Camilla Zanaglio, Nicola Polverelli, Elif Dereli Eke, Mariella D’Adda, Mirko Farina, Cristina Bucelli, Luigi Scaffidi, Eleonora Toffoletti, Clara Deambrogi, Fabio Stagno, Micaela Bergamaschi, Luca Franceschini, Elisabetta Abruzzese, Maria Domenica Divona, Marco Gobbi, Francesco Di Raimondo, Gianluca Gaidano, Mario Tiribelli, Massimiliano Bonifacio, Chiara Cattaneo, Alessandra Iurlo, Domenico Russo

**Affiliations:** ^1^ Unit of Blood Diseases and Stem Cell Transplantation, DPT of Clinical and Experimental Sciences University of Brescia, ASST Spedali Civili di Brescia Brescia Italy; ^2^ CREA Laboratory (Centro di Ricerca Emato‐Oncologica AIL) ASST Spedali Civili di Brescia Brescia Italy; ^3^ Division of Hematology ASST Spedali Civili of Brescia Brescia Italy; ^4^ Hematology Division Foundation IRCCS Ca' Granda‐Ospedale Maggiore Policlinico Milan Italy; ^5^ Department of Medicine, Section of Hematology University of Verona Verona Italy; ^6^ Division of Hematology and Bone Marrow Transplantation, Department of Medical Area University of Udine Udine Italy; ^7^ Division of Hematology, Department of Translational Medicine University of Eastern Piedmont Novara Italy; ^8^ Hematology Section and BMT Unit Rodolico Hospital A.O.U. Policlinico – V. Emanuele Catania Italy; ^9^ Clinical Hematology, Dipartimento Terapie Oncologiche Integrate Ospedale Policlinico San Martino Genova Italy; ^10^ Department of Biomedicine and Prevention The University Tor Vergata Rome Italy; ^11^ Division of Hematology S. Eugenio Hospital Roma Italy

**Keywords:** chronic myeloid leukemia, digital PCR (dPCR), minimal residual disease (MRD) monitoring, treatment‐free remission (TFR), tyrosine kinase inhibitors (TKI) discontinuation

## Abstract

Treatment‐free remission (TFR) by tyrosine kinase inhibitors (TKI) discontinuation in patients with deep molecular response (DMR) is a paramount goal in the current chronic myeloid leukemia (CML) therapeutic strategy. The best DMR level by real‐time quantitative PCR (RT‐qPCR) for TKI discontinuation is still a matter of debate. To compare the accuracy of digital PCR (dPCR) and RT‐qPCR for *BCR‐ABL1* transcript levels detection, 142 CML patients were monitored for a median time of 24 months. Digital PCR detected *BCR‐ABL1 *transcripts in the RT‐qPCR undetectable cases. The dPCR analysis of the samples, grouped by the MR classes, revealed a significant difference between MR^4.0^ and MR^4.5^ (*P* = 0.0104) or MR^5.0^ (*P* = 0.0032). The clinical and hematological characteristics of the patients grouped according to DMR classes (MR^4.0^ vs MR^4.5‐5.0^) were superimposable. Conversely, patients with dPCR values <0.468 *BCR‐ABL1* copies/µL (as we previously described) showed a longer DMR duration (*P* = 0.0220) and mainly belonged to MR^4.5‐5.0^ (*P* = 0.0442) classes compared to patients with higher dPCR values. Among the 142 patients, 111 (78%) discontinued the TKI treatment; among the 111 patients, 24 (22%) lost the MR^3.0 ^or MR^4.0^. RT‐qPCR was not able to discriminate patients with higher risk of MR loss after discontinuation (*P* = 0.8100). On the contrary, according to dPCR, 12/25 (48%) patients with *BCR‐ABL1* values ≥0.468 and 12/86 (14%) patients with *BCR‐ABL1* values <0.468 lost DMR in this cohort, respectively (*P* = 0.0003). Treatment‐free remission of patients who discontinued TKI with a dPCR <0.468 was significantly higher compared to patients with dPCR ≥ 0.468 (TFR at 2 years 83% vs 52% *P* = 0.0017, respectively). In conclusion, dPCR resulted in an improved recognition of stable DMR and of candidates to TKI discontinuation.

## 1 INTRODUCTION

Therapy with tyrosine kinase inhibitors (TKI) changed the fate of chronic myeloid leukemia (CML). Two decades after the introduction of imatinib (IM), the life expectancy of CML patients treated with TKI has approached to that of the general population^1^ and, now, the new objectives are focused on improving the management of the disease and, possibly, the quality of life of the patients.^2^, ^3^, ^4^

The current policy of CML therapy with TKI is to achieve a faster major molecular response (MMR or MR^3.0^ = *BCR‐ABL1/ABL1* IS ≤0.1%) to prevent the progression to blastic phase and to obtain a deep molecular response (DMR or MR^4.0^ if ≤0.01% *BCR‐ABL1/ABL1 *%IS, or MR^4.5^ if ≤0.0032% *BCR‐ABL1/ABL1 *%IS or MR^5.0^ if ≤0.001 *BCR‐ABL1/ABL1 *%IS) to gain the opportunity of a treatment discontinuation.^5^

The results from the Stop IMatinib (STIM) and TWISTER pivotal trials challenged the rule that TKI therapy should be continued lifelong. Since then, both IM^6^, ^7^, ^8^ and second‐generation TKI discontinuation studies^9^, ^10^ confirmed that about 50‐60% of patients with a DMR can successfully stop TKI, achieving a treatment‐free remission (TFR). These important clinical trials demonstrated that TKI discontinuation did not cause CML‐related deaths and, moreover, molecular relapses are still sensitive to therapy resumption.^11^, ^12^

As mentioned above, TFR is one of the most important objectives in CML patients, and recently, TKI discontinuation has become a reality also in clinical practice. In fact, in June 2017, the European Society of Medical Oncology (ESMO) introduced the option of TKI interruption out of the clinical trials too.^13^ However, patients’ inclusion criteria for discontinuation such as the prognostic score, the overall duration of TKI treatment, the “stable” DMR and the levels of DMR are still matters of debate. Therefore, the TKI discontinuation strategy cannot be considered as optimized in this setting. Clinically relevant questions were addressed also by the French CML Study Group, which recently published the recommendations on discontinuation of TKI in CML for clinical practice.^12^ They remark the importance of determining the best level of DMR for TKI discontinuation. Clinical evidences unexpectedly highlight the absence of a linear correlation between the depth of the DMR, quantified following the IS, and the TFR maintenance rate. One of the causes could be related to the intrinsic limitations of real‐time quantitative PCR (RT‐qPCR), particularly concerning its lack of precision, especially in the quantification of the low levels of the target, and the variation of its sensitivity from one test to another.^14^, ^15^

For these reasons, the great majority of patients who undergo TKI discontinuation frequently have a DMR with undetectable levels of *BCR‐ABL1* transcript by RT‐qPCR, Overall, according to the published data, 50%‐60% of patients with undetectable DMR by RT‐qPCR are expected to lose the DMR.^16^, ^17^, ^18^, ^19^

Therefore, the RT‐qPCR cannot be considered as an optimal tool neither to select the best candidates for treatment discontinuation nor to design personalized treatment programs, especially in the era of the more potent second‐generation TKI.

In the last years, the digital PCR (dPCR) has emerged as a more sensitive and accurate detection tool of minimal residual disease (MRD) and this increased the interest for its use in the clinical practice.^20^, ^21^, ^22^ The dPCR provides an absolute target sequence quantity, and recently, the alignment of the methods for *BCR‐ABL1* transcript quantification by using the Qx100/Qx200 Droplet Digital PCR System (Biorad) and the QuantStudio 3D Digital PCR System (Thermofisher) has been accomplished.^23^

Although the dPCR is not yet routinely applied for the standard analysis of molecular MRD in CML, preliminary data suggest that it is more sensitive and accurate than RT‐qPCR for monitoring the *BCR‐ABL1* transcript levels and, possibly, for predicting the patients who are going to relapse after discontinuation of TKI.^24^

This study focused on the MRD RT‐qPCR/dPCR comparative monitoring in 142 CML patients treated with TKI and with durable DMR (>2 years), as conventionally assessed by RT‐qPCR, before the enrollment.

The aim of this study was to evaluate the reliability and the efficiency of dPCR for a better evaluation of “stable” DMR and for a better selection of the candidates for treatment discontinuation.

## 2 METHODS

### 2.1 Patients

In total, 142 CML patients treated with TKIs (IM, nilotinib [NIL], or dasatinib [DAS]) for a median of 99 months (range 14‐215) and with durable (≥2 years) RT‐qPCR DMR (median 71 months; range 24‐171) were enrolled into the study, approved by the Ethical Committee of each participating Center. The patients were recruited by 10 Italian Hematologic Centers belonging to the CML GIMEMA (Gruppo Italiano Malattie Ematologiche dell'Adulto) Working Party. The clinical and hematological features of the patients, at the time of the enrollment, included: age, type of *BCR‐ABL1* transcript, Sokal risk distribution at diagnosis, the first‐ and second‐line TKI treatment, treatment dosage and duration, time to complete cytogenetic response (CCyR), MMR, DMR, and best molecular response (MR).

Table 1 reports the cohort's characteristics according to DMR class at the time of the enrollment into the study. Of the 142 cases, 116 (82%) had more than 24 months of DMR when they were enrolled and started (Time point 0) to be comparatively evaluated by the conventional RT‐qPCR and dPCR (RT‐qPCR/dPCR).

**Table 1:** Clinical and hematological characteristics of 142 Ph + CML patients with stable DMR comparatively monitored by RT‐qPCR and dPCR grouped in MR class by RT‐qPCR (MR^4.0^ vs MR^4.5‐5.0^) and by dPCR (≥ or <0.468 copies/µL) at enrollment

Variable		RT‐qPCR			dPCR		
	Total (n = 142)	MR^4.0^(n = 60) (42%)	MR^4.5‐5.0^ (n = 82) (58%)	*P*‐value	dPCR at enrollment ≥0.468 copies/µL (n = 31) (22%)	dPCR at enrollment <0.468 copies/µL (n = 111) (78%)	*P*‐value
M/F	82/60	36/24	46/36	0.6419	17/14	65/46	0.7108
Median age (range)	53 (20‐80) y	52.5 (22‐80) y	53 (20‐80) y	0.9763	53 (22‐77) y	53 (20‐80) y	0.6710
BCR‐ABL transcript at diagnosis							
B3A2	84 (59%)	35 (58%)	49 (60%)	0.6978	17 (55%)	67 (60%)	0.5731
B2A2	53 (37%)	24 (40%)	29 (35%)		14 (45%)	39 (35%)	
B2A2/B3A2	4 (3%)	1 (2%)	3 (4%)		—	4 (4%)	
NA	1 (1%)	—	1(1%)		—	1(1%)	
Sokal							
L	63 (44%)	24 (40%)	39 (48%)	0.5074	16 (52%)	47 (42%)	0.8358
I	52 (37%)	24 (40%)	28 (34%)		10 (32%)	42 (38%)	
H	22 (15%)	11 (18%)	11(13%)		4 (13%)	18 (16%)	
NA	5 (4%)	1 (2%)	4 (5%)		1 (3%)	4 (4%)	
1st line TKI							
IM	108 (76%)	49 (82%)	59 (72%)	0.4460	23 (74%)	85 (77%)	0.7372
NIL	28 (20%)	10 (17%)	18 (22%)		6 (19%)	22 (20%)	
DAS	5 (3.5%)	1 (1%)	4 (5%)		2 (7%)	3 (3%)	
BOS	1 (0.5%)	—	1 (1%)		—	1 (1%)	
1st TKI median dose (range)							
IM	400 (300‐800)	400 (400‐800)	400(300‐800)	1.0000	400 (400‐800)	400 (300‐800)	1.0000
NIL	400 (300‐800)	400 (400‐600)	400(300‐800)		400 (400‐600)	400 (300‐800)	
DAS	100 (100‐100)	100 (100‐100)	100(100‐100)		100 (100‐100)	100 (100‐100)	
BOS	500 (500‐500)	—	500 (500‐500)		—	500 (500‐500)	
Shift to second‐line TKI	31 (22%)	17 (28%)	14 (17%)	0.4664	7 (23%)	24 (22%)	0.8051
NIL	18 (58%)	9 (53%)	9 (64%)		5 (71%)	13 (54%)	
DAS	11 (36%)	7 (41%)	4 (29%)		2 (29%)	9 (38%)	
IM	1 (3%)	—	1 (7%)		—	1 (4%)	
BOS	1 (3%)	1 (6%)			—	1 (4%)	
Duration of TKIs treatment	99 (14‐215) m	98 (19‐215) m	100(14‐188) m	0.5272	99 (26‐199) m	99 (14‐215) m	0.7184
Time to CcgR							
3 mo	75 (53%)	34 (57%)	41 (41%)	0.1732	14 (45%)	61 (55%)	0.2819
6 mo	34 (24%)	14 (23%)	20 (24%)		9 (29%)	25 (23%)	
12 mo	12 (9%)	2 (3%)	10 (12%)		2 (7%)	10 (9%)	
NA	6 (4%)	4 (7%)	2 (2%)		3 (10%)	3 (3%)	
Time to MMR	6 (2‐112) m	6 (2‐112) m	6(2‐83) m	0.3879	6 (2‐40) m	6 (2‐112) m	0.9773
Time to DMR	12 (3‐120) m	23 (3‐120) m	12 (3‐119) m	0.2678	12 (3‐78) m	13 (3‐120) m	0.6211
Time to best MR	48 (2‐172) m	59 (2‐162) m	42 (5‐172) m	0.1762	53 (3‐140) m	46 (2‐172) m	0.9180
DMR duration (overall)	71 (24‐171) m	79 (24‐171) m	65 (24‐164) m	0.1314	50 (24‐144) m	76 (24‐171) m	***0.0220***
MR at first dPCR				—			
MR^4.0 ^total	60	—	—		18	42	***0.0442***
MR^4.0 ^undetectable	32 (42%)				10 (58%)	22 (38%)	
MR^4.5‐5.0 ^total	82				13	69	
MR^4.5‐5.0 ^undetectable	54 (58%)				11 (42%)	43 (62%)	

The times are expressed in years (y) or in months (mo). For RT‐qPCR and dPCR, the Mann‐Whitney and *t* test with Welch correction have been, respectively, used for comparison of the following continuous variables: age, duration of TKI treatment, time to MMR, time to DMR, time to best MR and DMR duration. A chi‐square analysis was performed for comparison of the following categorical variables: sex, BCR‐ABL transcript, Sokal class, 1st line TKI, TKI dose, shift to second‐line TKI, time to CcgR, MR t first dPCR. CML, chronic myeloid leukemia; DAS, dasatinib; NIL, nilotinib.

Bold and italic indicates the significance of the *P* value.

The patients were grouped into two pairs of DMR classes: the ones with MR^4.0^ vs the MR^4.5‐5.0^, and the ones with ≥0.468 vs those with <0.468 *BCR‐ABL1* copies/µL, according to the dPCR cutoff value of 0.468 *BCR‐ABL1* copies/µL, discriminating the deep molecular responders, as previously reported.^24^ A new receiver operating characteristic (ROC) curve analysis was carried out on the present cohort of patients and confirmed the reliability of the abovementioned cutoff in identification of the DMR patients by dPCR. (Figure S1).^24^

The assignment of DMR class, by using the RT‐qPCR values, was based on the results of the first RT‐qPCR evaluation, at the time of enrollment (Time point 0). In particular, at the Time point 0, according to RT‐qPCR results, 60 (42%) out of 142 patients had an MR^4.0^, and 32 (53%) out of 60 cases had MR^4.0^ undetectable; 82 (58%) had an MR^4.5^ or MR^5.0^ and 54 (66%) out of 82 cases were undetectable. On the other hand, according to dPCR results, 31 (22%) out of 142 patients had ≥0.468 *BCR‐ABL1* copies/µL, and 111 cases (78%) had <0.468 *BCR‐ABL1* copies/µL. (Table 1).

The median time of concomitant RT‐qPCR/dPCR monitoring was 24 months (range 12‐40). The median follow‐up of the entire cohort is 19 (1‐76).

Of note, 111 (78%) out of 142 patients discontinued the TKIs therapy. Fifty (45%) of patients who discontinued TKI therapy were enrolled in different discontinuing trails: ENESTFreedom (5/111, 4.5%), ENESTpath (8/111, 7.2%), and NP0 in Brescia (37/111, 33.3%).

Sixty‐one patients (55%) discontinued TKIs therapy out of clinical trials according to the Center policy based on clinical practice and shared patient's decision. All patients were evaluated at the time of discontinuation and after the discontinuation, while 13 cases were monitored also before the discontinuation of treatment (Table 2).

**Table 2:** Clinical and hematological characteristics of 111 patients who discontinued TKI treatment comparatively monitored by RT‐qPCR and dPCR grouped in MR class by RT‐qPCR (MR^4.0^ vs MR^4.5‐5.0^) and by dPCR (≥ or <0.468 copies/µL) at enrollment

Variable	Total (n = 111)	RT‐qPCR			dPCR		
		MR^4.0 ^(n = 45) (41%)	MR^4.5‐5.0 ^(n = 66) (59%)	P‐value	dPCR at enrollment ≥0.468 copies/µL (n = 25) (23%)	dPCR at enrollment <0.468 copies/µL (n = 86) (77%)	P‐value
M/F	61/50	26/19	35/31	0.6216	12/13	49/37	0.4271
Median age (range)	53 (20‐80) y	52 (22‐80) y	54 (20‐80) y	0.7331	50 (22‐77) y	53 (20‐80) y	0.5752
BCR‐ABL transcript at diagnosis							
B3A2	83 (75%)	34 (76%)	49 (74%)	0.7603	17 (68%)	66 (77%)	0.3038
B2A2	23 (21%)	10 (22%)	13 (20%)		8 (32%)	15 (17%)	
B2A2/B3A2	4 (4%)	1 (2%)	3 (5%)		—	4 (5%)	
NA	1 (1%)	—	1 (1%)		—	1 (1%)	
Sokal							
L	45 (41%)	16 (36%)	29 (44%)	0.6781	13 (52%)	32 (37%)	0.5870
I	43 (39%)	20 (44%)	23 (35%)		8 (32%)	35(41%)	
H	19 (5%)	8 (18%)	11 (17%)		3 (12%)	16(19%)	
NA	4 (4%)	1 (2%)	3 (4%)		1 (4%)	3 (3%)	
1st line TKI							
IM	86 (77%)19	37 (77%)	49 (74.2%)	0.5792	19 (76%)	67 (78%)	0.7531
NIL	(17%)	7 (20%)	12 (18.2%)		4 (16%)	15 (17%)	
DAS	5 (5%)	1 (3%)	4 (6.1%)		2 (8%)	3 (4%)	
BOS	1 (1%)	—	1 (1.5%)		—	1 (1%)	
1st TKI median dose (range)							
IM	400 (400‐800)	400 (400‐800)	400 (400‐800)	1.0000	400 (400‐800)	400 (400‐800)	1.0000
NIL	400 (400‐600)	400 (400‐600)	400 (400‐600)		400 (400‐400)	400 (400‐600)	
DAS	100 (100‐100)	100 (100‐100)	100 (100‐100)		100 (100‐100)	100 (100‐100)	
BOS	500 (500‐500)	—	500 (500‐500)		—	500 (500‐500)	
Shift to second‐line TKI	21 (19%)	10 (22%)	11 (17%)	0.3512	4 (16%)	17 (20%)	0.7591
NIL	11 (52%)	4 (64%)	7 (64%)		3 (75%)	8 (47%)	
DAS	8 (38%)	5 (50%)	3 (45%)		1 (25%)	7 (41%)	
IM	1 (5%)	—	1 (1%)			1 (6%)	
BOS	1 (5%)	1 (10%)	—			1 (6%)	
Duration of TKIs treatment	97 (33‐194) m	97 (36‐194) m	95 (33‐162) m	0.6018	99 (38‐163) m	96 (33‐194) m	0.7031
Time to CcgR							
3 mo	64 (58%)	29 (64%)	35 (53%)	0.4549	12 (43%)	52 (60%)	0.2458
6 mo	27 (24%)	11 (24%)	16 (24%)		9 (39%)	18 (21%)	
12 mo	7 (6%)	1 (2%)	6 (9%)		1 (4%)	6 (7%)	
NA	4 (4%)	2 (4%)	2 (3%)		2 (9%)	2 (2%)	
Time to MMR	7 (2‐112) m	8 (2‐112) m	6 (2‐83) m	0.2510	6 (2‐40) m	8 (2‐112) m	0.6201
Time to DMR	19 (3‐120) m	28 (3‐120) m	14 (3‐119) m	0.1502	16 (3‐78) m	19 (3‐120) m	0.3889
Time to best MR	52 (3‐162) m	78 (3‐162) m	46 (5‐144) m	0.1672	50 (3‐83) m	52 (5‐162) m	0.5940
DMR duration (overall)	74 (5‐162) m	81 (5‐154) m	66 (24‐162) m	0.050	54 (5‐144) m	76 (24‐162) m	***0.0250***
DMR duration (until discontinuation)	50 (9‐116) m	57 (10‐116) m	45 (9‐113) m	0.1388	45 (10‐108) m	52 (9‐116) m	0.7272
MR at first dPCR							
MR^4.0 ^total	45 (41%)	45 (100%)	0 (0%)	—	12 (48%)	33 (38%)	0.3881
MR^4.0 ^undetectable		23 (51%)			6 (50%)	17 (52%)	
MR^4.5‐5.0 ^total	66 (59%)	0 (0%)	66 (100%)		13 (52%)	53 (62%)	
MR^4.5‐5.0 ^undetectable			45 (68%)		11 (85%)	34 (64%)	
Patients who lost MR^3.0^ or MR^4.0^	24 (22%)	9 (20%)	15 (23%)	0.8100	12 (48%)	12 (14%)	***0.0003***
Median time	3 (1‐19) m	3 (1‐4) m	5 (1‐19) m	0.1371	3 (1‐19) m	4 (1‐16) m	0.6381
lost MR^3.0^		8/9 (67%)	2/15 (13%)		5/12 (42%)	3/12 (25%)	
lost MR^4.0 ^or 1 Log		1/9 (33%)	13/15 (87%)		7/12 (58%)	9/12 (75%)	

The times are expressed in years (y) or in months (mo). For RT‐qPCR and dPCR, the Mann‐Whitney and *t* test with Welch correction have been, respectively, used for comparison of the following continuous variables: age, duration of TKI treatment, time to MMR, time to DMR, time to best MR, DMR duration, and number of patients who lost MR. A chi‐square analysis was performed for comparison of the following categorical variables: sex, BCR‐ABL transcript, Sokal class, 1st line TKI, TKI dose, shift to second‐line TKI, time to CcgR, MR at first dPCR, and time to loss of MR. DAS, dasatinib; IM, imatinib; NIL, nilotinib.

Bold and italic indicates the significance of the *P* value.

During the monitoring period, a total of 556 peripheral blood samples were comparatively analyzed by RT‐qPCR/dPCR.^17^, ^24^, ^25^

The study was performed according to good clinical practice and Helsinki's Declaration, and the patients gave their informed consent.

### 2.2 RT‐qPCR and dPCR analyses

Conventional RT‐qPCR measurements were carried out at the Reference Laboratory of each participating Center, according to ELN Guidelines.^4^, ^25^, ^26^, ^27^ At each time point scheduled for the MRD monitoring, 10 mL of peripheral blood was sampled and used for RT‐qPCR analysis.^17^, ^24^

Molecular responses by RT‐qPCR were defined according to the latest laboratory recommendations and using *ABL1* as a housekeeping gene.^24^ Measurable MR was assigned following the international scale (IS) and scored MR^4.0^ if ≤0.01% *BCR‐ABL1 *%IS, MR^4.5^ if ≤0.0032% *BCR‐ABL1 *%IS, and MR^5.0^ if ≤0.001 *BCR‐ABL1 *%IS. Minimum sum of *ABL1* reference gene transcripts, irrespective of whether *BCR‐ABL1* was detected or not, 10.000, 32.000, and 100.000 for MR^4.0^, MR^4.5^, and MR^5.0^, respectively.^24^

The participating reference laboratories belonged the Gimema Labnet and accredited by the Gimema Labnet Quality Committee to release the results of RT‐qPCR analysis, since certified for the quantification of *BCR‐ABL1* according to the IS, as recommended by the International Experts Panel's Guide Lines.^25^ These analysis were performed as detailed in Data S2.

To the purpose of our study, the loss of the MR was defined as loss of MR^3.0 ^according to ENESTFreedom and ENESTpath criteria (13 cases) or as “at least two positive RT‐PCR results showing a significant increase (by 10 times; ie, one log), at two consecutive assessments or loss of major molecular response (MMR)” according to what reported by STIM study (98 cases).^6^The dPCR analysis was performed on the same cDNA samples used for RT‐qPCR, in order to limit the retrotranscription step variability.^28^ Therefore, each patient enrolled in the study was comparatively analyzed for the *BCR‐ABL1* transcript by RT‐qPCR and by dPCR at every time point.

The dPCR analysis was centralized at the Laboratory CREA (Centro di Ricerca Emato‐Oncologico AIL) of the ASST Spedali Civili of Brescia and performed as previously described.^24^

Briefly, the cDNA was quantified using a Quant‐iT™ OliGreen® ssDNA kit (Thermofisher Scientific) by Infinite200 (Tecan) and diluted at 50 ng/µL since this quantity resulted the best to obtain a deep quantification and to avoid the saturation of the chip.

We prepared 16 μL of reaction mix containing 8 μL of 2X QuantStudio 3D Digital PCR Master Mix (Thermofisher Scientific), 0.8 μL of 20X TaqMan‐MGB‐FAM‐probe assay, 1.1 μL of diluted cDNA, and 6.1 μL of nuclease‐free water (Qiagen). The negative control reaction mix contained 8 µL of 2X QuantStudio 3D Digital PCR Master Mix, 0.8 μL of 20X TaqMan‐MGB‐FAM‐probe assay, and of 7.2 μL nuclease‐free water. One negative control was loaded every thermal cycling run containing samples prepared with the same mix. The reverse transcription negative control reaction mix contained 8 μL of 2X QuantStudio 3D Digital PCR Master Mix, 0.8 μL of 20X TaqMan‐MGB‐FAM‐probe assay, 1.2 μL of reverse transcription blank, and 6.1 μL of nuclease‐free water.

For each sample, we loaded 15 μL of the reaction mixes onto a QuantStudio 3D Digital PCR 20K Chip (Thermofisher Scientific) using the automatic chip loader and the signal was amplified by the following thermocycling profile: 95°C for 8 minutes, 45 cycles at 95°C for 15 seconds, and 60°C for 1 minute, with a final extension step at 60°C for 2 minutes. The amplification was followed by the chips imaging and the secondary analysis performed with the QuantStudio 3D AnalysisSuite Cloud Software. All samples were analyzed twice by different operators and the final results were expressed as means of the number of *BCR‐ABL1* copies/µL of reaction of the replicates.^24^

### 2.3 Statistical analysis

Clinical and laboratory data were categorized as continuous or categorical variables. The *t* test or Mann‐Whitney and chi‐square were used to compare the continuous and categorical variables, respectively. The *t* test with Welch's correction has been used in the evaluation of dPCR subgroups as unbalanced sample sizes had to be taken into account (in fact 111 and 31 patients in the overall cohort, and 86 and 25 patients in the discontinuation cohort presented dPCR lower and higher than 0.468, respectively). Normality distribution of variables was assessed by a Shapiro‐Wilk test. The type of analysis performed is stated in figures’ and tables’ legends.

Treatment‐free remission was calculated from the date of TKI withdrawal to the date of loss of MR^3.0 ^or MR^4.0 ^or last control by the Kaplan‐Meier method. Comparison of subgroups was carried out by the log‐rank test. Univariate Cox regression analysis was used to evaluate the association of variables with TFR. Variables included age at diagnosis, sex, previous therapy with IFN, time to MMR and DMR, Sokal class, type of transcript, time to discontinuation, use of frontline second‐ generation TKIs, MR classes considering detectable and undetectable transcript, and dPCR. Multivariable analysis has been performed on dPCR values and, by univariate analysis, the duration of DMR resulted significant.

Receiver operator curves analysis was done to recalculate the optimal dPCR cutoff value to discriminate the patients with DMR and lower probability to lose MR^3.0^ after TKI discontinuation. Notably, a cutoff of 0.468 was found to be coincidental with the previous one identified discriminating the deep molecular responders by dPCR.^24^ This value divided patients with lower risk of disease molecular relapse better, with an area under the curve of 0.69 (CI 95% 0.57‐0.81), specificity of 85% and sensitivity of 54% (Figure S1). All p‐values are two‐sided and significance level has been set below 0.05. Statistical analyses were conducted with EZR software (version 1.33), as previously described.^29^

## 3 RESULTS

This study aimed to comparatively evaluate the efficiency and the reliability of the RT‐qPCR and dPCR for the recognition of an accurate and “stable” DMR and selection of candidates to TKIs discontinuation.

For that purpose, 142 CML patients with a durable RT‐qPCR DMR (≥2 years) were comparatively monitored by RT‐qPCR/dPCR every 3‐4 months for a median time of 24 months (range 12‐40), from the time of enrollment (first RT‐qPCR/dPCR analysis = Time point 0) thereafter (last follow‐up). Furthermore, 111/142 patients underwent treatment discontinuation and were valuable for TFR.

First, the levels of *BCR‐ABL1* minimal residual disease, as conventionally assessed by RT‐qPCR, were correlated with the dPCR values measured at the same time points.

**Figure 1:**
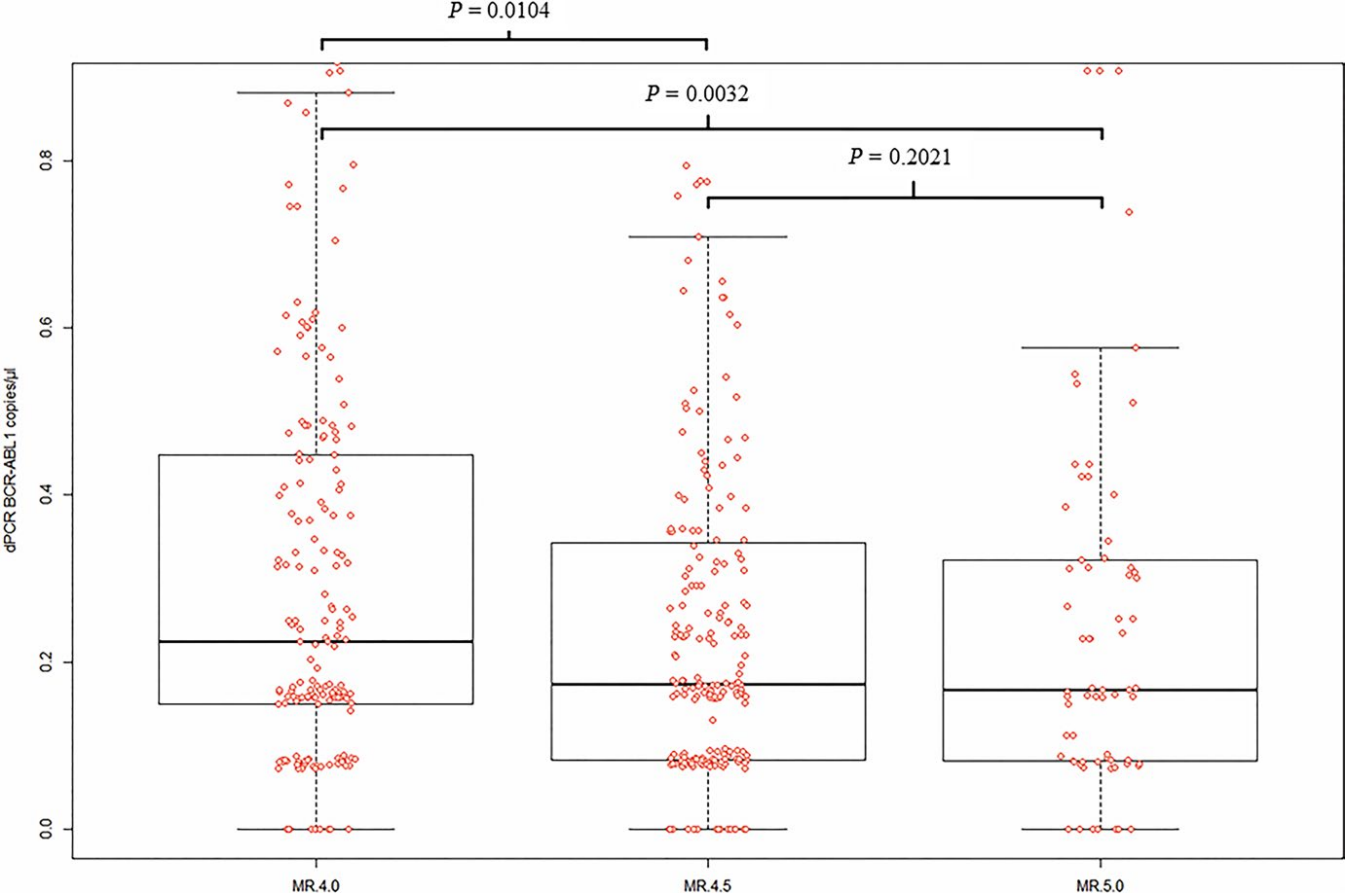
Levels of *BCR‐ABL1* transcript measured by dPCR (y‐axis) according to different MR classes calculated by RT‐qPCR (x‐axis). RT‐qPCR *BCR‐ABL1* results were grouped into MR^4.0^, MR^4.5^, and MR^5.0 ^class (“X axis”). When the same samples were analyzed by dPCR, different levels of BCR‐ABL1 copies were measured (“Y axis”). The difference was statistically significant between the MR^4.0^ class and the MR^4.5^ (*P* = 0.01) or the MR^5.0^ class (*P* = 0.003), but not between the MR^4.5^ and the MR^5.0^ classes (*P* = 0.2). The statistic test used was *t* test. In box and whiskers plot, center line represents the median values; the boxes’ limits represent the lower and the higher quartile; whiskers define the extreme values; and points represent the single analysis values

The 556 samples comparatively analyzed by RT‐qPCR and dPCR are plotted in Figure 1. RT‐qPCR *BCR‐ABL1* results were grouped into MR^4.0^, MR^4.5^, and MR^5.0^class (“X axis”). When the same samples were analyzed by dPCR, different levels of *BCR‐ABL1* copies were measured (“Y axis”). The numbers of *BCR‐ABL1* copies/µL in each MR class resulted heterogeneous and widely dispersed, even the relative medians and range were progressively decreasing moving from the MR^4.0^ class to MR^5.0^class. The difference was statistically significant between the MR^4.0^ class and the MR^4.5^ (*P* = 0.0104) or the MR^5.0^ class (*P* = 0.0032), but not between the MR^4.5^ and the MR^5.0^ classes (*P* = 0.2021) (Figure 1).

**Figure 2:**
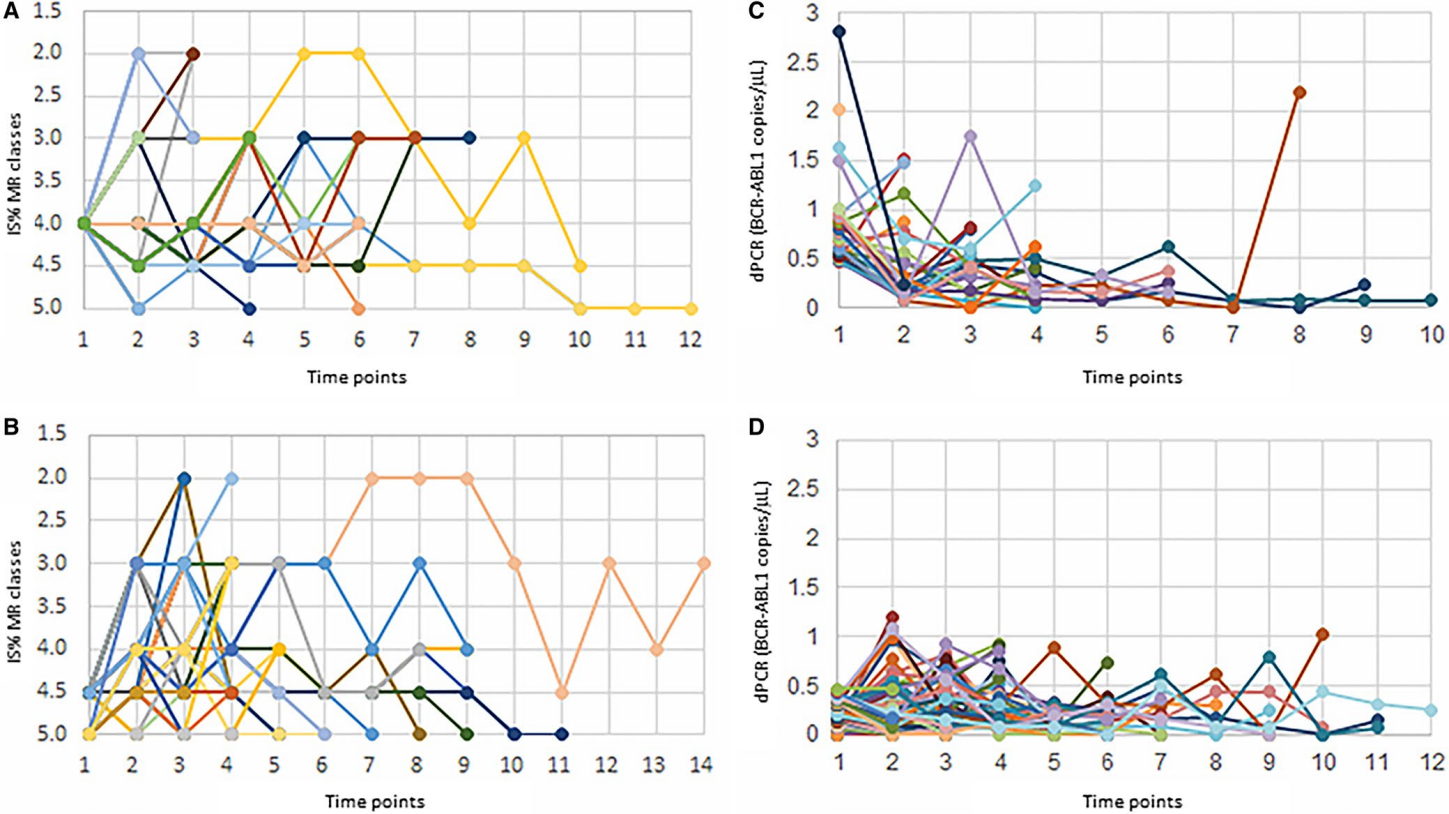
Molecular residual disease (MRD) over time as measured by RT‐qPCR and dPCR. (A) MRD monitoring by RT‐qPCR in patients with MR^4.0^ at Time Point 0. (B) MRD monitoring by RT‐qPCR in patients with MR^4.5‐5.0^ at Time Point 0. (C) MRD monitoring by dPCR in patients with value of *BCR‐ABL1* copies/µL ≥0.468 at Time Point 0. (D) MRD monitoring by dPCR in patients with value of *BCR‐ABL1* copies/µL <0.468 at Time Point 0

Then, we comparatively analyzed the course of RT‐qPCR monitoring in the MR^4.0^ and MR^4.5‐5.0^ patients, as well as the course of molecular dPCR monitoring in the patients with ≥ and <0.468 *BCR‐ABL1* copies/µL (Figure 2).

The patients grouped into the MR^4.0 ^and MR^4.5‐5.0^ class by RT‐qPCR started from the same MR determined at Time point 0 (Figure 2A,B); on the other hand, patients with dPCR ≥0.468 *BCR‐ABL1* copies/µL, at the Time point 0, presented different levels of molecular MRD and had a median value of 0.758 (range 0.469‐2.806), while patients with dPCR <0.468 copies/µL presented lower levels of *BCR‐ABL1* copies and their median value was of 0.171 (range 0‐0.467) (Figure 2C,D).

No difference between the monitoring course of MR^4.0 ^and MR^4.5‐5.0^ classes could be appreciated and measured. Instead, observing the course of dPCR MRD molecular monitoring, the patients belonging the group below the cutoff of <0.468 *BCR‐ABL1* copies/µL seemed to show lower and more “stable” levels of MRD along all the time of monitoring (Figure 2D). During the follow‐up, patients with dPCR <0.468 *BCR‐ABL1* copies/µL at the Time point 0, presented a median value of 0.175 copies/µL (range 0‐1.348), while patients with dPCR ≥0.468 copies/µL presented higher levels of *BCR‐ABL1* copies and had a median value of 0.393 copies/µL (range 0‐2.195). The differences at Time point 0 and the variations during the follow‐up period did not result statistically significant between the two groups.

In order to evaluate the correlation between the DMR, the characteristics of patients, and the probability to maintain the TFR, the analysis was carried out first on all of the 142 cases and then in the patients who discontinued the treatment (n = 111), grouped into two pairs of classes according to the RT‐qPCR and dPCR results at Time point 0: the MR^4.0^ vs MR^4.5‐5.0^ class, and ≥0.468 vs <0.468 *BCR‐ABL1* copies/µL group (Tables 1 and 2).

The clinical and hematological characteristics of the patients grouped according to DMR classes (M^4.0^ vs MR^4.5‐5.0^) were superimposable, while the patients with dPCR <0.468 had a longer DMR duration (*P* = 0.0250) and more frequently belonged to MR^4.5‐5.0^ (*P* = 0.0442) classes compared to patients with higher dPCR values (Table 1).

Overall, 111/142 (78%) patients discontinued the TKI treatment after a median time of 97 months of TKI therapy (range 33‐194). MRD levels assessed by RT‐qPCR at the time of treatment discontinuation identified 45/111 (41%) patients with MR^4.0^ (23/45—51%—presented *BCR‐ABL1* transcript undetectable) and 66/111 (59%) with MR^4.5‐5.0^ (45/66—68%—presented *BCR‐ABL1* transcript undetectable). These patient groups, MR^4.0^ vs MR^4.5‐5.0^, did not show any significant statistical difference in clinical and hematological characteristics (Table 2).

By dPCR, 25/111 (23%) had ≥0.468 and 86/111 (77%) had <0.468. No significant statistical difference was observed in clinical and hematological characteristics, except for a longer overall DMR duration in patients with dPCR <0.468 (76 vs 54 months. *P* = 0.0250) (Table 2).

**Figure 3:**
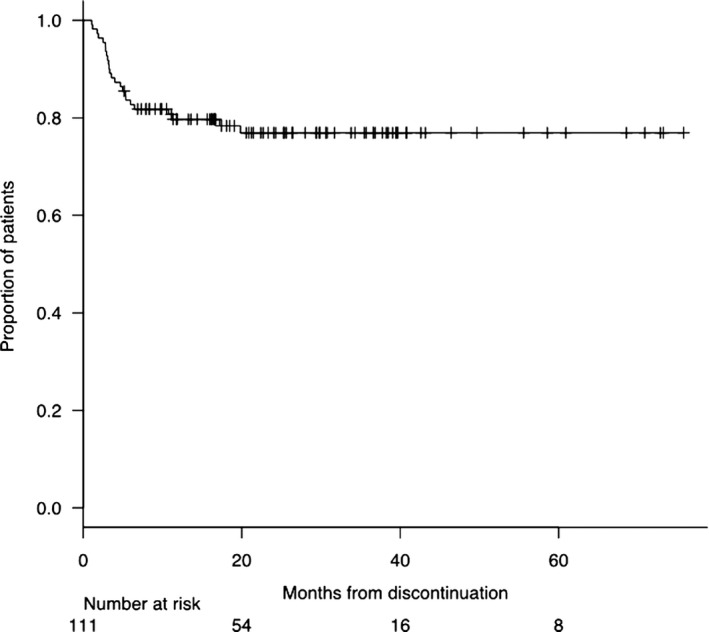
Treatment‐free remission (TFR) curve of 111 patients in deep molecular response (DMR) who discontinued TKI. The probability of maintaining TFR was 83%, 80%, and 77% at 6, 12, and 24 months, respectively. The plot has been performed by the Kaplan‐Meier method

At last follow‐up, 24/111 (22%) patients lost the MR^3.0^ (10/24) or the MR^4.0^ (14/24) (Table 2). The median time of MR loss was 3 months from TKI discontinuation (range 1‐19), with an incidence rate of 10.4 pt/year. Overall, the TFR was 83% at 6 months, 80% at 12 months, and 77% at 24 months (Figure 3). According to RT‐qPCR at the time of TKI discontinuation, 9/45 were in MR^4.0^ (20%) and 15/66 (23%) were in MR^4.5‐5.0^ (*P* = 0.8100); according to dPCR, 12/25 (48%) and 12/86 (14%) had *BCR‐ABL1* values ≥0.468 and <0.468, respectively (*P* = 0.0003) (Table 2).

**Figure 4:**
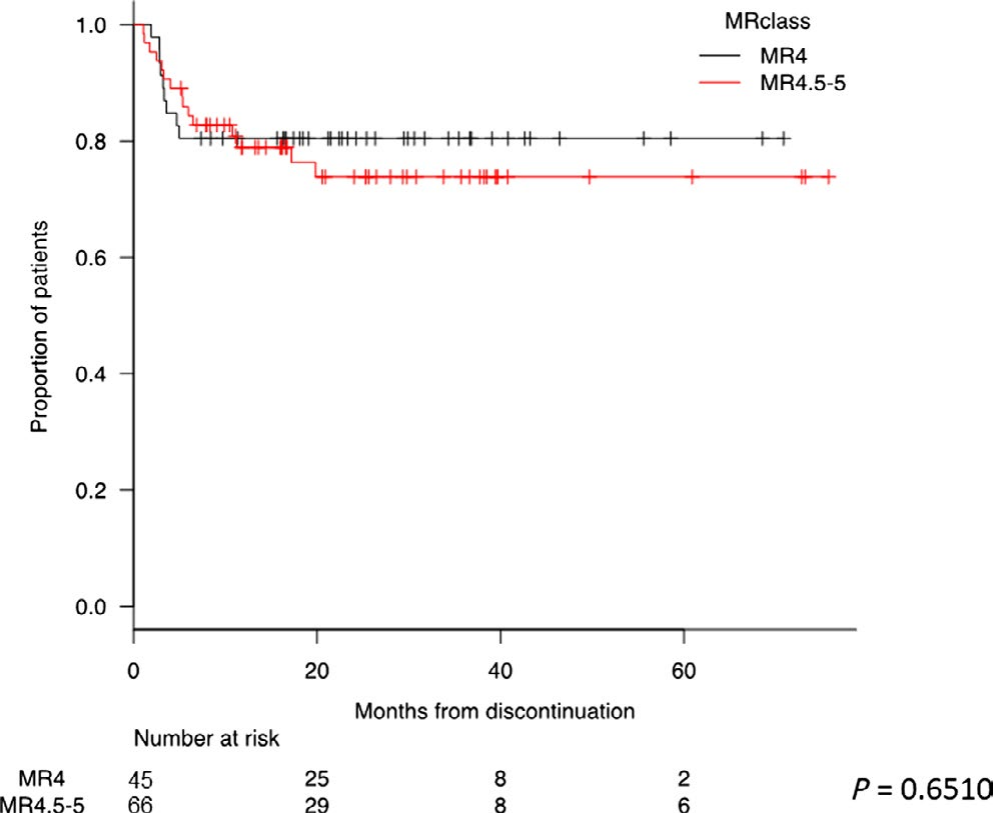
Treatment‐free remission (TFR) curves according to MR class measured by RT‐qPCR at the time of discontinuation. The red curve represents patients discontinued with MR^4.0^, and the black curve patients with MR^4.5‐5.0^. The probability of maintaining TFR for patients discontinued with MR^4.0^ was 80% at both 1 and 2 years. The probability of maintaining TFR for patients discontinued with MR^4.5‐5.0^ was 79% and 74% at 1 and 2 years, respectively. Kaplan‐Meier analysis was used for the evaluation of TFR. Comparison of subgroups was carried out by the log‐rank test

**Figure 5:**
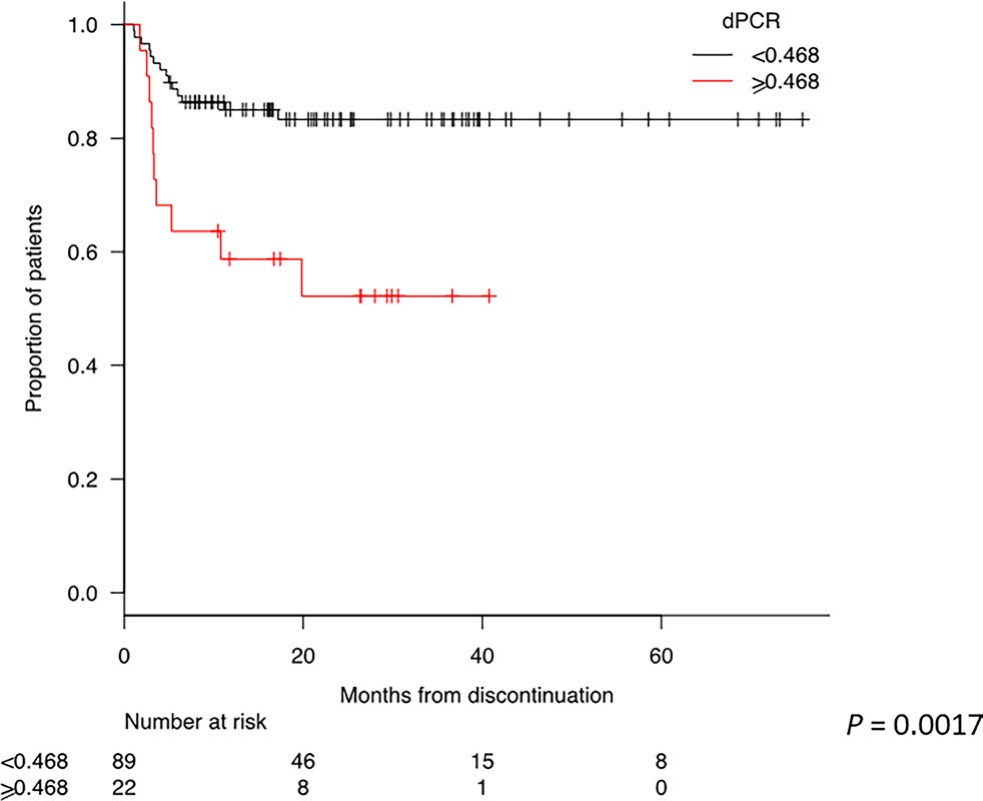
Treatment‐free remission (TFR) curves according to dPCR values. The red curve represents patients discontinued with a dPCR value lower than 0.468 and the black curve patients with a dPCR value higher than 0.468. The probability of maintaining TFR for patients discontinued with dPCR <0.468 was 85% and 83% at 1 and 2 years, respectively. The probability of maintaining TFR for patients discontinued with dPCR ≥ 0.468 was 59% and 52% at 1 and 2 years, respectively. A Kaplan‐Meier analysis was used for the evaluation of TFR. Comparison of subgroups was carried out by a log‐rank test

By survival analysis, no statistical significant differences according to different MR classes were found (Figure 4; *P* = 0.6510). An additional Kaplan‐Meier analysis was carried out on detectable MR^4.0^ vs total MR^4.5‐5.0 ^(Supplementary File 3A), on MR^4.0‐4.5 ^detectable and total MR^5.0^ (Figure S2B), and on MR^4.0 ^detectable with MR^4.5 ^detectable and MR^5.0 ^at all (Figure S2C). In any cases, the consideration of the detectability of the transcript was not statistically significant. On the contrary, a dPCR value below 0.468 *BCR‐ABL1* copies/µL at the time of discontinuation highly stratified patients with lower probability to lose MR Indeed, the TFR at 1 and 2 years was 85% and 83% patients presenting dPCR values below the defined cutoff compared to 59% and 52% of patients with higher dPCR values at the time of discontinuation, respectively (*P* = 0.0017) (Figure 5).

**Figure 6:**
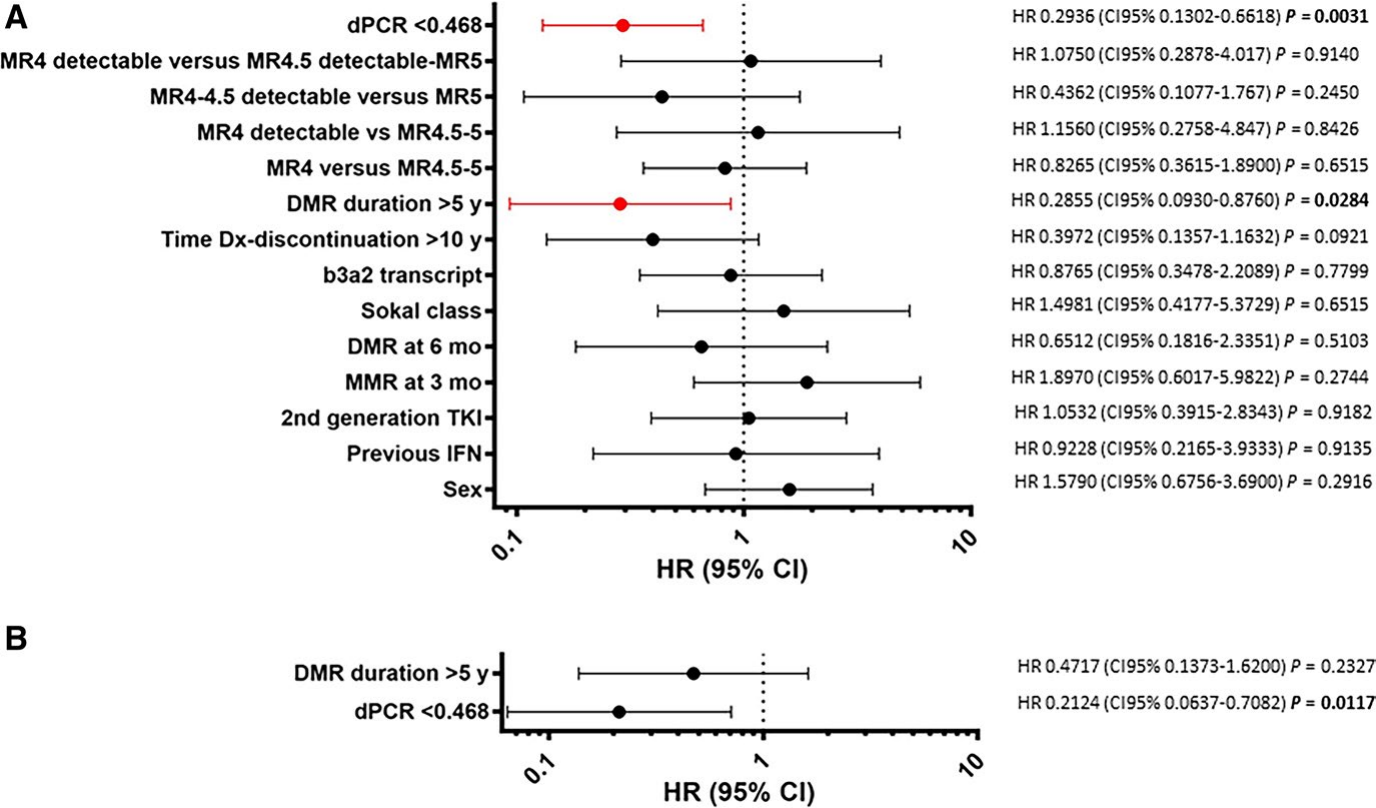
Univariate and multivariate analyses for the prediction of treatment‐free remission (TFR). (A) Univariate Cox regression analysis. Variables included were age at diagnosis, sex, previous therapy with IFN, time to MMR and DMR, Sokal class, type of transcript, time to discontinuation, use of frontline second‐generation TKIs, MR classes considering detectable and undetectable transcript, and dPCR. DMR duration > 5 years (HR 0.2855, CI 95% 0.0931‐0.8760, *P* = 0.0284) and dPCR (HR 0.2936, CI 95% 0.1302‐0.6618, *P* = 0.0031) resulted significantly predictable of TFR maintenance. (B) Multivariate analysis—only dPCR retained its significant value (HR 0.2124, CI 95% 0.0637‐0.7082, *P* = 0.0117)

The univariate and multivariate analyses confirmed the predictive value of dPCR. In particular, DMR duration > 5 years (HR 0.2855, CI 95% 0.0931‐0.8760, *P* = 0.0284) and dPCR (HR 0.2936, CI 95% 0.1302‐0.6618, *P* = 0.0031) resulted significantly predictable of TFR maintenance in univariate analysis. By multivariate analysis, only dPCR retained its significant value (HR 0.2124, CI 95% 0.0637‐0.7082, *P* = 0.0117 (Figure 6).

In 13 cases, a comparative monitoring of DMR by RT‐qPCR/dPCR was possible for at least 24 months before TKIs discontinuation. In these cases, 10 out of 13 (77%) had stable <0.468 *BCR‐ABL1* copies/µL by dPCR and 2/10 (20%) lost MR. On the other hand, 3/13 (23%) presented stable ≥0.468 *BCR‐ABL1* copies/µL by dPCR and 2/3 (67%) lost MR (*P* = 0.1245).

## 4 DISCUSSION

This study, based on a multicentric cohort of CML patients with durable DMR (≥2 years), shows that the dPCR appears to be more accurate and sensitive than conventional RT‐qPCR for detecting and monitoring the *BCR‐ABL1* molecular levels. Moreover, dPCR is potentially able to improve the recognition of DMR and the selection of the candidates to TKI treatment discontinuation.

The study reports on a longlasting comparative RT‐qPCR/dPCR monitoring of *BCR‐ABL1* transcript levels in 142 CML patients having a median duration of TKI treatment and DMR of 8.3 years and 5.9 years, respectively. These long periods could explain why a very high TFR rate (80% at 1 year) was observed in our cohort of patients, in comparison to an expected rate of 50%‐60%, usually reported in the literature.^6^, ^7^ Notably, the TKI exposure and DMR duration was 4.8 and 3 years in STIM trial, 6 and 3.3 years in IFN‐IM cohort of Twister trial, and 6.3 and 2.4 in STOP 2G‐TKI study, respectively. Durable DMR, more than the duration of TKI treatment, is considered the most important modifiable prognostic factor playing a key role for TKI discontinuation success, but at present, there is no general agreement on the duration either of the TKIs therapy or of DMR. 11

Indeed, ESMO recommends at least 5 years of TKI therapy combined with ≥2 years of DMR before TKI discontinuation,^13^ while the French CML Study Group requires at least 2 years of MR^4.5^ before discontinuation in order to reach the TFR. Moreover, a great uncertainty still remains for the level of MR.^12^

Considering the pivotal clinical trials concerning TKI discontinuation, in EURO‐SKI study, a threshold of 3.1 years of MR^4.0^ was identified as dissecting the patients with the lowest probabilities to lose the TFR. 30 In the JALSG‐STIM213 trial, patients in MR^5.0^ undetectable before IM or nilotinib discontinuation had significantly higher probabilities to maintain the TFR than those in MR^4.5^. 31, 32 In the STIM study, the following duration of the treatment was considered: at least 3 years of IM was required, with at least 2 years of MR^4.5^ undetectable levels of *BCR‐ABL1* quantified following the IS system. 7

It clearly appears that the sensitivity and the detectability of *BCR‐ABL1* transcript are additional elements that may play an important role. In fact, because of its low sensitivity, RT‐qPCR could not discriminate the depth of response in patients with undetectable *BCR‐ABL1* transcript and a patient with undetectable MR^4.0^ could belong to MR^5.0^.

In our experience, the majority of the samples classified as DMR had an undetectable level of transcript, confirming the low RT‐qPCR capability to detect low levels of MRD while the dPCR appeared to overcome this important bias thanks to its high sensitivity and accuracy, allowing a better selection of patients eligible for TKI discontinuation. Our data, according to Takahashi and colleagues,^31^ encourage TFR studies using dPCR, offering better quantitative accuracy than RT‐qPCR IS for DMR.

Looking at our RT‐qPCR and dPCR comparative analysis and monitoring, dPCR allowed to highlight a high heterogeneity of MRD levels in patients belonging the same class of MR at the Time point 0. Indeed, measuring the absolute number of *BCR‐ABL1* copies/µL by dPCR, it was evident that both the patients with MR^4.0^ and those with MR^4.5‐5.0^ had different levels of MRD (Figure 1). Importantly, absolute numbers of *BCR‐ABL1* copies/µL could be detected and measured also in MR^4.0^, MR^4.5^, or MR^5.0^ patients with undetectable *BCR‐ABL1* transcripts by RT‐qPCR.

Furthermore, due to a sort of “normalizing” effect of RT‐qPCR, the courses of the patients monitored by RT‐qPCR did not allow the identification of patients with “stable” DMR from the patients with the “unstable” DMR, neither in the group of MR^4.0^ responses nor in the group of MR^4.5‐5.0^ responses, independently from a detectable or undetectable *BCR‐ABL1* transcript.

Observing MRD courses by dPCR in the group of patients with ≥ or <0.468 BCR‐ABL1 copies/µL, it was possible to appreciate a specific trend for each patient. Moreover, patients presenting MRD levels <0.468 *BCR‐ABL1* copies/µL at Time point 0, apparently showed lower levels of transcript along all the follow‐up than patients with MRD levels ≥0.468 *BCR‐ABL1* copies/µL, even if we did not find any statistical difference between median levels of ≥ and <0.468 *BCR‐ABL1* copies/µL groups. Lacking of a statistically significant difference could be due to the narrow range of values measured by dPCR and to the needing of a dPCR power test, focused on this end point and carried out on a larger cohort of CML patients.

These data suggest that the dPCR may be able to identify a set of optimal and stable molecular responders based on the levels and the stability of MRD (<0.468 *BCR‐ABL1* copies/µL).

One hundred and eleven patients discontinued the TKI treatment and 24 (22%) of them lost the MR^3.0 ^or the MR^4.0 ^according to the criteria reported by Etienne et al.^6^

In our cohort of patients, the TFR rate was higher than the one currently reported in the IM and second‐generation TKI discontinuation studies^31^, ^32^ but, as expected, the loss of MR occurred in 24/111 (22%) cases early after TKIs discontinuation (3 months). This highly positive selection could be explained, as above reported, by the prolonged treatment and the DMR duration.^30^, ^31^ Furthermore, the major part of the patients (75%) who underwent TKIs discontinuation was characterized by the presence of the transcript b3a2. This transcript has been recently associated with a better response to the TKIs treatment, resulting in higher sustained DMR rate,^39^ and a longer TFR.^40^It has to be underlined that considering our entire cohort, patients with <0.468 dPCR values of MRD had also a significantly longer duration of DMR and this seemed to significantly translate in a lower rate of loss of MR, either MR^3.0 ^or MR^4.0^.

By the way, the rate of MR^3.0 ^or MR^4.0^ loss in the cases with <0.468 *BCR‐ABL1* copies/µL by dPCR resulted significantly lower (14%) than one of the cases with ≥0.468 *BCR‐ABL1* copies/µL (48%). Indeed, the probability to predict the TFR was 86% for the cases with <0.468 *BCR‐ABL1* copies/µL and 52% for the cases with ≥0.468 *BCR‐ABL1* copies/µL, and this difference was statistically significant (*P*=0.0003) (Table 2).

Looking at the clinical and hematological characteristics of these two groups of patients, they resulted comparable, except for the longer duration of DMR (*P* = 0.025).

Interestingly, these data first showed a potential correlation between the duration and stability of DMR and a measurable reduction of molecular levels of *BCR‐ABL1* transcript. In our opinion, they suggest the value <0.468 *BCR‐ABL1* copies/µL of transcript by dPCR would reflect the threshold of *BCR‐ABL1 *transcript suitable for a higher probability of treatment discontinuation success. In fact, in our study, the dPCR resulted the only parameter associated with TFR by multivariate analysis (Figure 6), while no statistically significant difference (*P* = 0.6510) was observed between the rate of MR loss of patients with MR^4.0^ (detectable or undetectable) vs MR^4.5‐5.0^ (detectable or undetectable) (Figure 4).

There is general agreement on considering RT‐qPCR not sufficiently adequate to measure low levels of *BCR‐ABL1* transcripts and this has to be considered particularly relevant in the era of the more potent second‐generation TKIs. 25, 38 However, while everybody knows the limits of RT‐qPCR, newly available technologies have not been yet introduced in the quantification of the MRD, particularly in the setting of deep responders CML patients, possible candidates to TKIs discontinuation.

In order to routinely apply dPCR for *BCR‐ABL1* detection and quantification for a personalized management of CML patients, other efforts have to be performed in the standardization of the procedures and in the alignment of the results generated by the different dPCR platforms.^22^, ^24^, ^41^

No firm conclusion can be drawn and, certainly, the higher accuracy, sensitivity, and predictive value of dPCR have to be prospectively confirmed on a larger cohort of cases. However, this study practically tested the potential of dPCR and highlighted that this technique is able to detect measurable quantities of *BCR‐ABL1* transcripts in the cases with undetectable DMR by RT‐qPCR; that patients belonging to the same MR class do not have the same quantities of *BCR‐ABL1* transcript when measured by dPCR; and that dPCR may improve the recognition of “stable” DMR, hence contributing to a better selection of candidates to TKIs discontinuation.

## CONFLICT OF INTEREST

The authors declare no competing financial interests.

## AUTHOR CONTRIBUTIONS

DR, SB, and MM designed the study; SB, CZ, and EDE performed the dPCR analysis; MM, MF, MD, CC, AI, CB, M. Bonifacio, ET, MT, CD, GG, FS, FDR, M. Bergamaschi, MG, LF, MDD, and EA enrolled the patients and collected the clinical data; NP and EDE performed the statistical analysis; SB, MM, NP, EDE, CZ, and DR wrote the manuscript.
