# Supplementary figures and images for: Digital PCR improves the quantitation of DMR and the selection of CML candidates to TKIs discontinuation
Source: Cancer Med. 2019 Apr 4;8(5):2041–55. doi: 10.1002/cam4.2087 (PMC6536984; doi:10.1002/cam4.2087)

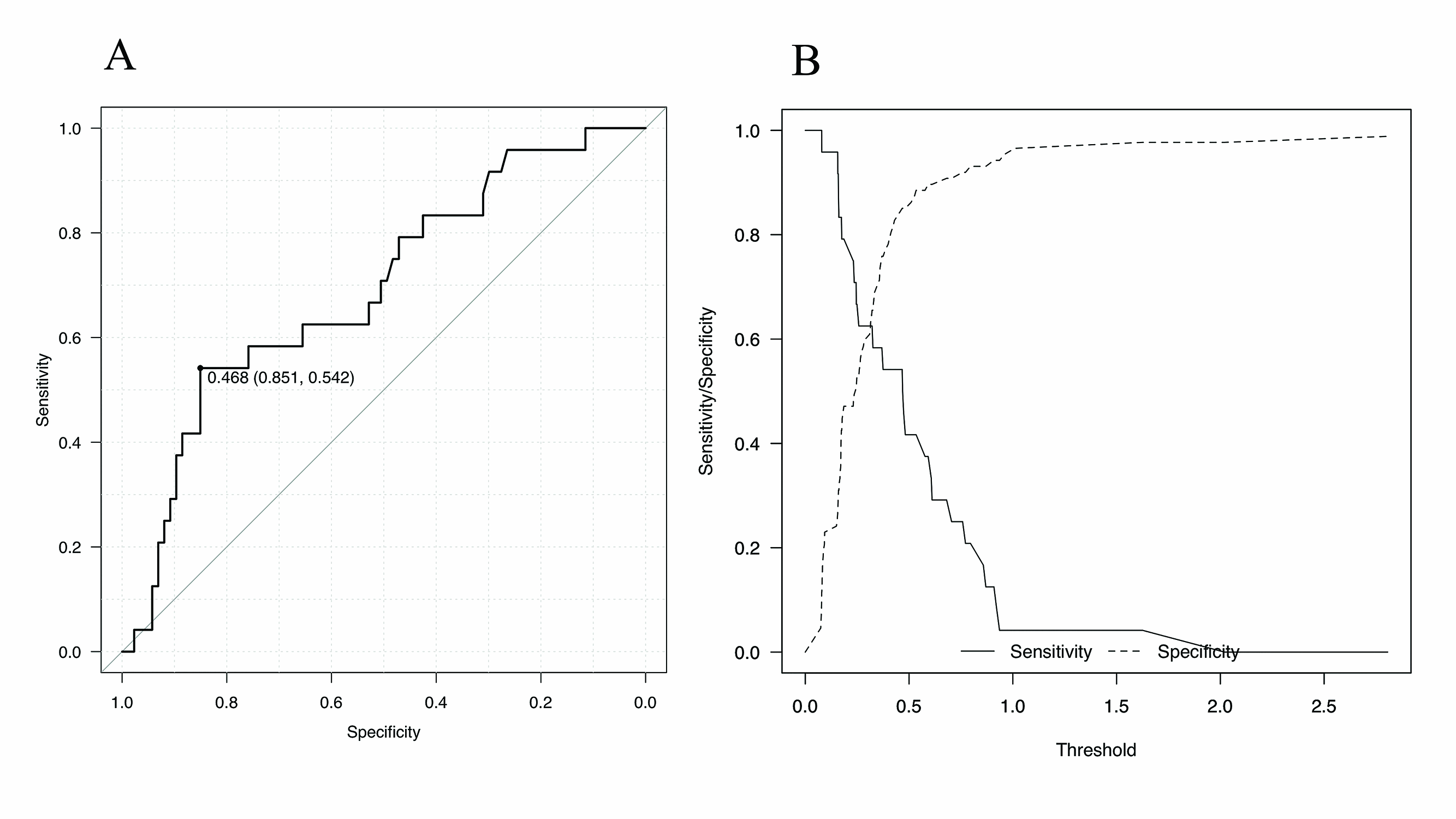

Supplement: Supplementary file 1 [file CAM4-8-2041-s001.tif]
