# Supplementary material for: Digital PCR improves the quantitation of DMR and the selection of CML candidates to TKIs discontinuation
Source: Cancer Med. 2019 Apr 4;8(5):2041–55. doi: 10.1002/cam4.2087 (PMC6536984; doi:10.1002/cam4.2087)
Supplement: Supplementary file 2 [file CAM4-8-2041-s002.docx]

**Supplementary 2**

The RNA extraction was performed either using automatized extractor Qiacube (Qiagen) or manually by NucleoSpin RNA plus (Machery Nagel), following the manifactures’ instructions. Both these extraction procedures were approved for a clinical diagnostic application in Italy.

The RT-PCR primers used by the different laboratories were the ones published by the European against cancer (EAC) program as endorsed by the European Leukemia Net (ELN)^28^. All the Laboratories performed the RT-qPCR reaction using ABI Prism (7000 or 7500) by Thermofisher and used ABL as reference gene.

The assays sequences were the following:

BCR-ABL1 assay

FW Primer TCCGCTGACCATCAAYAAGGA

Rev Primer CACTCAGACCCTGAGGCTCAA

Probe CCCTTCAGCGGCCAGTAGCATCTGA

ABL1 assay

FW Primer ACTCTAAGCATAACTAAAGG

Rev Primer GATGTAGTTGCTTGGGACCCA

Probe AAGCCCAAACCAAAAATThe retro-transcription and the RT-qPCR details are briefly here reported:

RT reaction

1 μg of total RNA in 10 μl of DNA- RNA-free H2O was retrotranscribed starting with an incubation at 70°C for 10’, followed by ice incubation. Then, 100U of Reverse transcriptase (Superscript I or II), 1 mM of dNTP, 10 mM of DTT, 25 μM of Random hexamers, 20U of RNAse inhibitor and RT buffer (according to the enzyme used) were added to a final volume of 20 μl. This step was followed by three subsequent incubations: at room temperature for 10’, 42°C for 45’, 99°C for 3’ with a final holding at 4°C.

RT-qPCR reaction

The RT-qPCR mix was obtained with 5 μl of final cDNA (retrotranscribed as described), 300 nM of each primer and 200 nM of probe mixed together in a unique assay, 12.5 μl of Master Mix, for a final volume of 25 μl. The thermalcycling profile was: 50°C for 2’, 95°C for 10’, followed by 95°C for 15’’ and 60°C for 1’ for 50 cycles.

The RT-qPCR reaction protocol was the same for BCR-ABL1 and ABL gene. Three BCR-ABL1 replicates and two ABL replicates were analyzed in order to obtain the raw data for the determination of the MRD following the IS.

The analysis method was 2^-ΔΔCt based and the Ct range was 34.5-43.8 for BCR-ABL1 and 22.9-25.7 for ABL.

The calculation of the positivity based on the number of Ct followed the last International guidelines relied by the panel of experts in 2015 (Cross N et al, Leukemia 2015)^26^.
